# Effects of the combination of anti-PcrV antibody and bacteriophage therapy in a mouse model of *Pseudomonas aeruginosa* pneumonia

**DOI:** 10.1128/spectrum.01781-24

**Published:** 2024-10-23

**Authors:** Ohara Junya, Fujiki Jumpei, Mao Kinoshita, Kazuki Sudo, Ken Kawaguchi, Keita Inoue, Yoshifumi Naito, Kiyoshi Moriyama, Tomohiro Nakamura, Hidetomo Iwano, Teiji Sawa

**Affiliations:** 1 Department of Anesthesiology, Graduate School of Medical Science, Kyoto Prefectural University of Medicine, Kyoto, Japan; 2 Laboratory of Veterinary Biochemistry, School of Veterinary Medicine, Rakuno Gakuen University, Ebetsu, Hokkaido, Japan; 3 Division of Critical Care Medicine, University Hospital, Kyoto Prefectural University of Medicine, Kyoto, Japan; 4 Department of Anesthesiology, School of Medicine, Kyorin University, Mitaka, Japan; Emory University School of Medicine, Atlanta, Georgia, USA

**Keywords:** bacteriophage therapy, PcrV, *Pseudomonas aeruginosa*, acute lung injury, type III secretion system

## Abstract

Acute lung injury caused by *Pseudomonas aeruginosa* is attributed to the translocation of cytotoxin into pulmonary epithelial cells via the *P. aeruginosa* type III secretion system. This virulence can be blocked with a specific antibody against PcrV in this secretion system. However, because anti-PcrV antibodies do not have bactericidal activity, the treatment of bacteria depends on the phagocytic system of the host. In this study, we investigated the therapeutic effect of combination therapy with an anti-PcrV antibody and bactericidal bacteriophages on acute lung injury and subsequent death in mice compared with a single treatment. After the mice intratracheally received a lethal dose of the cytotoxic *P. aeruginosa* strain, a second instillation was performed with saline, anti-PcrV IgG, bacteriophages, or a mixture of anti-PcrV and bacteriophages. The survival rates 24 h after infection were as follows: 7.1% in the saline group, 26.7% in the anti-PcrV group, 41.2% in the phage group, and 66.7% in the anti-PcrV + phage group (*P* < 0.001 vs saline-treated group). The activity of surviving mice in the anti-PcrV + phage group was significantly greater than that in the saline group. The lung weight in the anti-PcrV + phage group was significantly lower than that in the anti-PcrV group. In conclusion, combination therapy with an anti-PcrV antibody and a bacteriophage reduces acute lung injury and suggests improved survival compared with each treatment alone. This combination therapy, which does not rely on conventional antibiotics, could constitute a new strategy for treating multidrug-resistant *P. aeruginosa* infections.

**IMPORTANCE:**

Combination therapy with either bacteriophages alone or in combination with anti-PcrV antibodies in a mouse model of *Pseudomonas aeruginosa* pneumonia may reduce the acute lung injury and improve survival. This combination therapy, which does not rely on conventional antibiotics, may be a new strategy to treat multidrug-resistant *Pseudomonas aeruginosa* infections.

## INTRODUCTION

*Pseudomonas aeruginosa* is a Gram-negative bacillus that causes pneumonia in immunocompromised patients as an opportunistic infection. In recent years, the emergence of multidrug-resistant *P. aeruginosa* has made treating this disease difficult, and treatment using drugs other than anti-bacterial drugs has attracted increased amounts of attention (1, 2). Therefore, some new treatment methods that do not rely on conventional anti-bacterial drug therapy, such as antibody therapy and bacteriophage therapy, are needed (3–5). Since Twort discovered bacteriophages in 1915 (6), bacteriophage therapy was initially expected to be a new treatment for bacterial infections. However, this therapy did not spread outside Eastern European countries at that time, owing to the rise of penicillin and other anti-bacterial drugs. With the emergence of multidrug-resistant bacteria in recent years, phage therapy has become a limited therapeutic method that is also effective against drug-resistant bacteria. We previously isolated the *Pseudomonas* virus ΦR18, which showed more than 80% infectivity against 29 *P*. *aeruginosa* veterinary isolates (7). We found that phage therapy using ΦR18 was effective in a mouse model of bacterial equine keratitis, resulting in the complete prevention of keratitis caused by the clinical isolate of *P. aeruginosa* after administering the phage within 3 h of infection.

*P. aeruginosa* causes acute lung injury through the type III secretion system (8, 9). A specific antibody against PcrV, which is the cap protein of the secretory apparatus of the type III secretion system, suppresses the toxicity of the type III secretion system of *P. aeruginosa* (10), but the antibody itself is not bactericidal. However, bacteriophages act specifically on bacteria to induce bacteriolysis. The disadvantage of bacteriophages is that they require a certain amount of time for bacteriolysis because phage invasion and multiplication within the bacteria are needed. PcrV antibody therapy and phage therapy can overcome each other’s weaknesses when combined. Therefore, this study investigated the efficacy of combination therapy with an anti-PcrV antibody and a bacteriophage in a mouse model of *P. aeruginosa* pneumonia.

## RESULTS

### Lytic activity of the *Pseudomonas* virus ΦR18 against *P. aeruginosa* PA103

**Fig 1:**
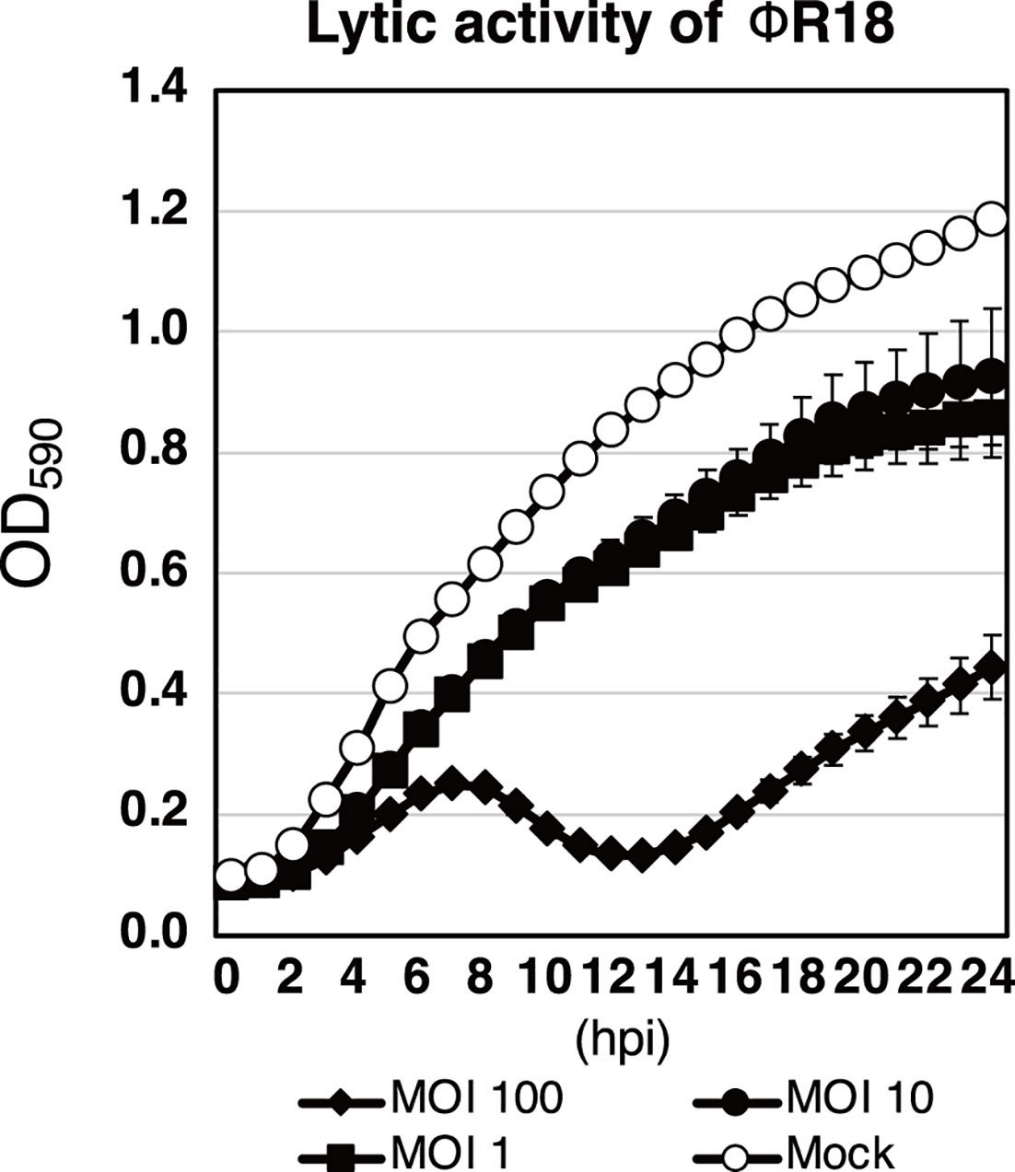
Lytic effects of ΦR18 on PA103. The lytic activity of ΦR18 was monitored for 24 h. The virus ΦR18 infected *P. aeruginosa* PA103 at MOIs of 1, 10, and 100. Mock indicates no phages added. The OD_590_ values represent the mean ± SD from three independent replicates.

We initially assessed the lytic activity of ΦR18, which is a podovirus in the classical morphological classification belonging to *Caudoviricetes*, *Kochitakasuvirus*. This podovirus has potential applications in phage therapy against the *P. aeruginosa* PA103 strain (7). At lower multiplicities of infection (MOIs) of 1 and 10, ΦR18 showed no efficient lytic activity against PA103 (Fig. 1). However, at a higher MOI of 100, ΦR18 showed efficient lytic activity, resulting in the suppression of PA103 growth from 0 to 8 h post-infection and a decrease in optical density (OD) 590 values from 8 to 13 h post-infection. The phage may achieve sufficient bacteriolysis approximately 8 h after infection. Therefore, during the first few hours, antibodies may inhibit the virulence of PA103 before bacteriolysis occurs.

### *P. aeruginosa* infection challenge in mice

The mice were divided into five groups (Table 1). Except for the uninfected control group, in the four infected groups of mice (see below for details), a solution containing PA103 (1.0 × 10^6^ CFU in 60 µL of saline) was instilled into the lungs of each mouse through an endotracheal needle, as described previously under light inhalational anesthesia using sevoflurane (11). As we reported previously, mice that received a lethal dose of PA103 (1.0 × 10^6^ CFU) became hypothermic within 4 h (12). The hypothermic pathology and mortality within 24 h in this mouse model of pneumonia is not a result of bacterial multiplication in the infected organ but rather a consequence of necrosis of lung epithelial cells. This necrosis was due to the translocation of the type III secretory toxin, ExoU, of *P. aeruginosa* into lung epithelial cells within 4 h after infection (13, 14). We also reported that the necrosis of lung epithelial cells due to ExoU toxin translocation occurs within 4 h after infection in an *in vitro* culture cell model (12). In addition, this pathology has been reported to disappear after infection with isogenic mutant strains lacking the enzymatically active ExoU of *P. aeruginosa* (13–15). Therefore, our therapeutic model focuses on the first 4–24 h after infection.

**TABLE 1:** Experimental groups

Group	*n*	Treatment
Saline uninfected	5	None
Infected with *P. aeruginosa* PA103		
Saline	14	Saline alone 30 µL
Anti-PcrV	15	Anti-PcrV IgG 1 µg, in 30 µL of saline
Phage	17	Bacteriophage ΦR18 4.0 × 10^7^ PFU,^*a*^ in 30 µL of saline
Anti-PcrV and phage	15	Anti-PcrV 1 µg and bacteriophage ΦR18 4.0 × 10^7^ PFU, in 30 µL of saline

^*a*^ PFU, plaque-forming unit.

**Fig 2:**
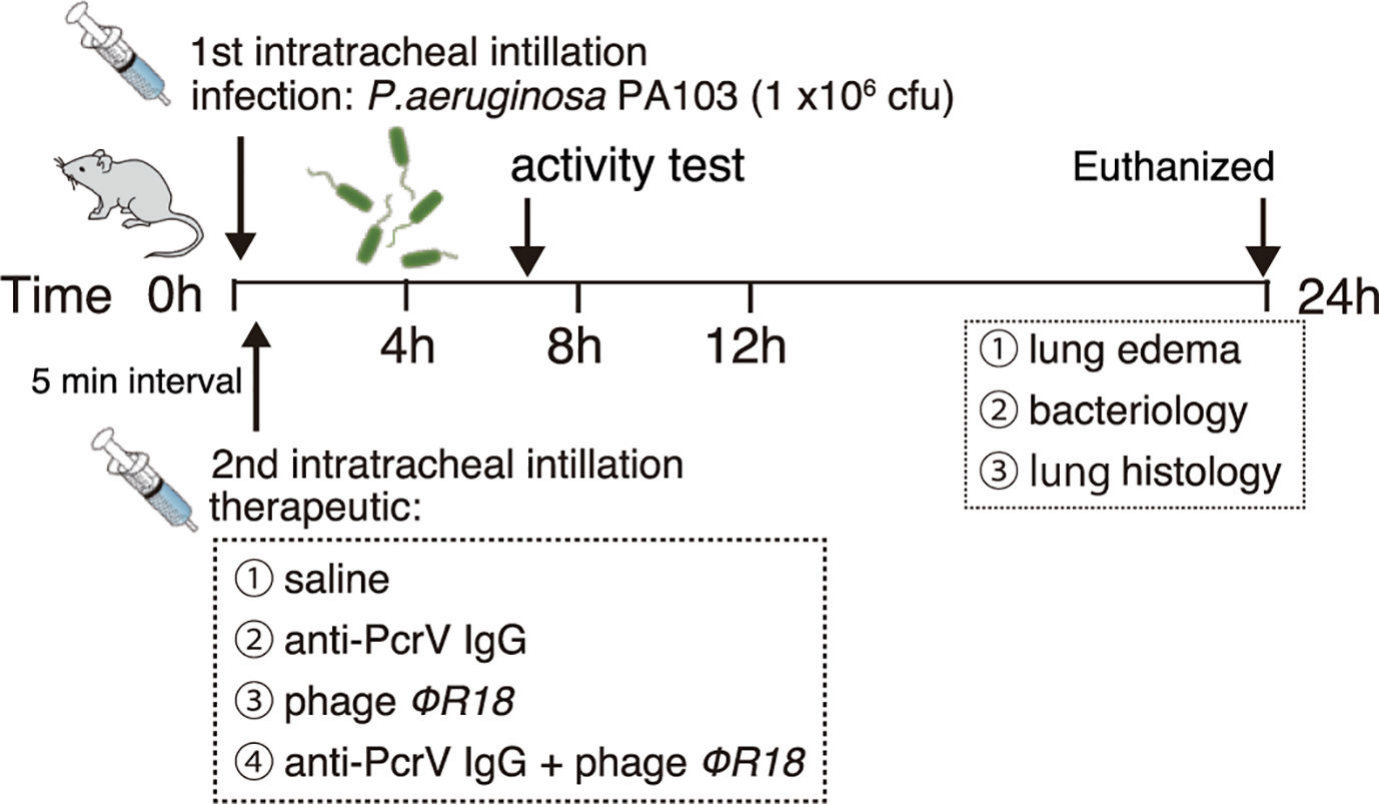
Experimental protocol.

The experimental protocol is shown in Fig. 2. Mice were intratracheally administered saline (60 µL) alone (uninfected control group) or *P. aeruginosa* PA103 (infected groups). Within 30 s after the first instillation, the mice were allowed to recover from inhalational anesthesia. The mice were anesthetized again 5 min after the first instillation, and a second instillation was performed on each infected mouse with 30 µL of solution containing saline (saline group), anti-PcrV IgG (1 µg in 30 µL of saline), bacteriophage solution (4.0 × 10^7^ PFU, phage group), or a mixture of anti-PcrV IgG (1 µg) and bacteriophage (4.0 × 10^7^ PFU) in 30 µL of saline (anti-PcrV + phage group). Mice in the uninfected control group were intratracheally administered saline (30 µL) again. After recovering from the second general anesthesia session in the cages, the infected mice were allowed to move freely, and the survival rate and body temperature were measured over the next 24 h.

### Body temperature and survival rate of infected mice

**Fig 3:**
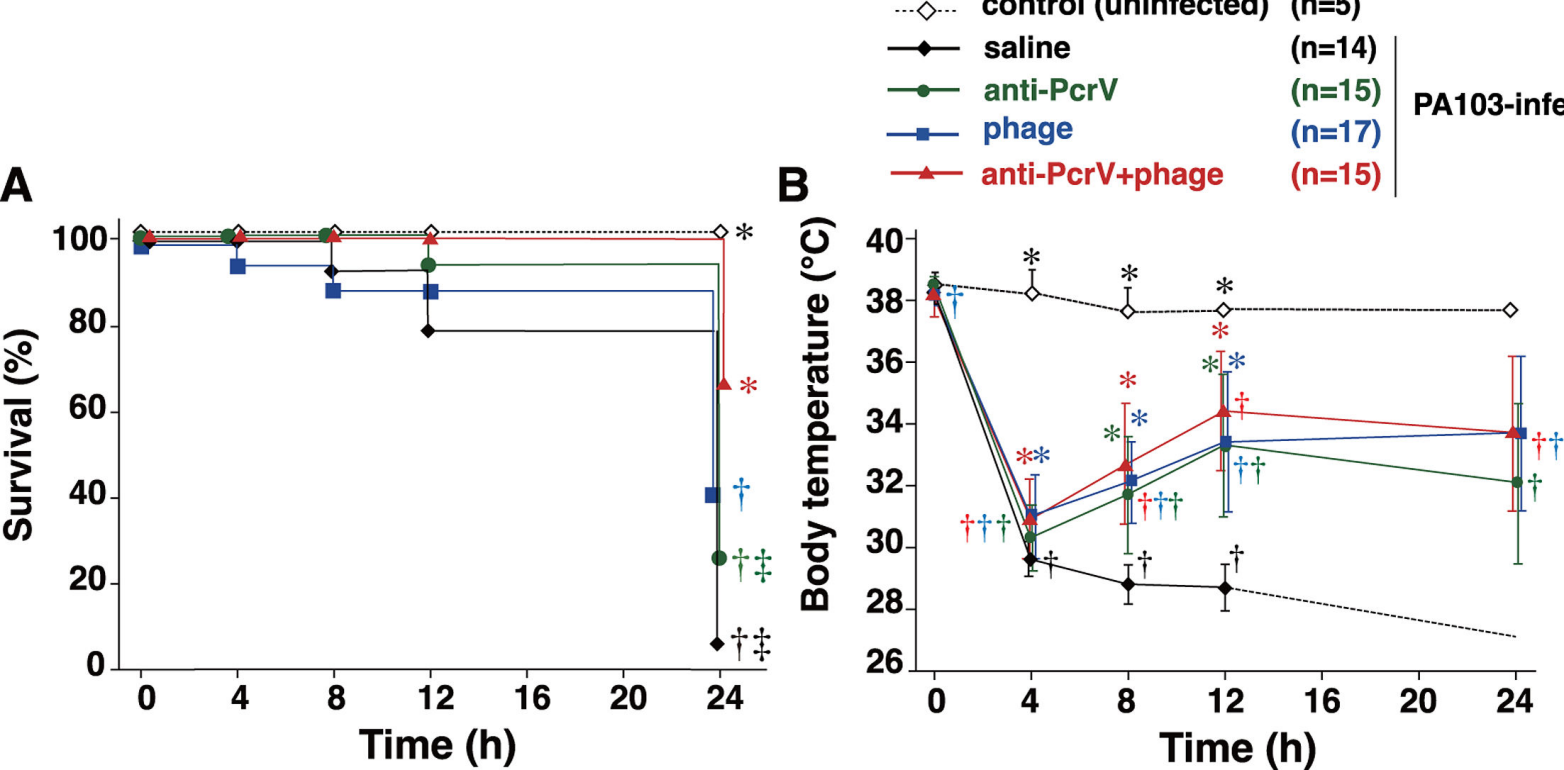
Survival and body temperature over time in mice infected with *P. aeruginosa* PA103. The mice were divided into four groups that received the first instillation (either saline or a lethal dose of *P. aeruginosa* PA103) or the second instillation (either saline, anti-PcrV IgG, bacteriophages, or anti-PcrV IgG plus bacteriophages). (A) The survival rates and body temperature were monitored for 24 h after intratracheal infection with *P. aeruginosa*. (B) The time course of body temperature for 24 h in mice infected with *P. aeruginosa*. The data are presented as the mean ± SD. *P < 0.05 compared with the saline group, †*P* < 0.05 compared with the uninfected control group, ‡*P* < 0.05 compared with the anti-PcrV + phage group.

Survival and body temperature were compared among the five groups of mice after 24 h (Fig. 3; Table 2). Except for mice in the uninfected control group, all mice intratracheally infected with PA103 (1.0 × 10^6^ CFU) became hypothermic and less active within 4 h. Any treatment, either anti-PcrV IgG, phage, or both, induced partial recovery from hypothermia and activity loss in some of the mice. After 24 h, the survival rates of the infected groups of mice were as follows: 7.1% in the saline group, 26.7% in the anti-PcrV group, 41.2% in the phage group, and 66.7% in the anti-PcrV + phage group (Fig. 3A). The survival rate was significantly greater in the anti-PcrV + phage group than in the saline group (*P* = 0.01) and in the anti-PcrV group (*P* = 0.025). The body temperature (mean ± SD) of surviving mice after *P. aeruginosa* administration in the saline group decreased from 38.3°C ± 0.4°C at 0 h to 32.2°C ± 0.4°C at 4 h, 28.8°C ± 0.6°C at 8 h, and 28.7°C ± 0.7°C at 12 h, and only one mouse survived with severe hypothermia at 27.1°C for 24 h (Fig. 3B). The body temperature in the surviving mice in the anti-PcrV group decreased from 38.5°C ± 0.3°C at 0 h to 30.3°C ± 1.0°C at 4 h and 31.7°C ± 1.8°C at 8 h. However, after 12 h, the body temperature recovered to 33.3°C ± 2.7°C at 12 h and 32.1°C ± 2.2°C at 24 h. The body temperature in the surviving mice in the phage group decreased from 38.2°C ± 0.4°C at 0 h to 31.0°C ± 1.3°C at 4 h and 32.1°C ± 1.3°C at 8 h. However, after 12 h, the body temperature recovered to 33.4°C ± 2.2°C at 12 h and 33.7°C ± 2.3°C at 24 h. The body temperature in the surviving mice in the anti-PcrV + phage group decreased from 38.1°C ± 0.6°C at 0 h to 30.9°C ± 1.3°C at 4 h and 32.7°C ± 1.9°C at 8 h. However, after 12 h, the body temperature recovered to 34.4°C ± 1.9°C at 12 h and 33.7°C ± 2.4°C at 24 h.

**TABLE 2:** Statistics of survival studies

Group		Survival rate [survivors/total number (%)]	*P* values		
			vs uninfected	vs untreated	vs anti-PcrV and phage
Uninfected	Saline	5/5 (100)	–	0.002^*a*^	0.146
Infected with *P. aeruginosa*	Saline (untreated)	1/14 (7.1)	0.002^*b*^	–	0.010^*c*^
	Anti-PcrV	4/15 (26.7)	0.007^*b*^	0.579	0.025^*c*^
	Phage	7/17 (41.2)	0.031^*b*^	0.33	0.114
	Anti-PcrV and phage	10/15 (66.7)	0.146	<0.010^*a*^	–

^*a*^ *P* < 0.05 compared with the saline group.

^*b*^ *P* < 0.05 compared with the uninfected control group.

^*c*^ *P* < 0.05 compared with the anti-PcrV + phage group.

### Activity in *P. aeruginosa-*infected mice

**Fig 4:**
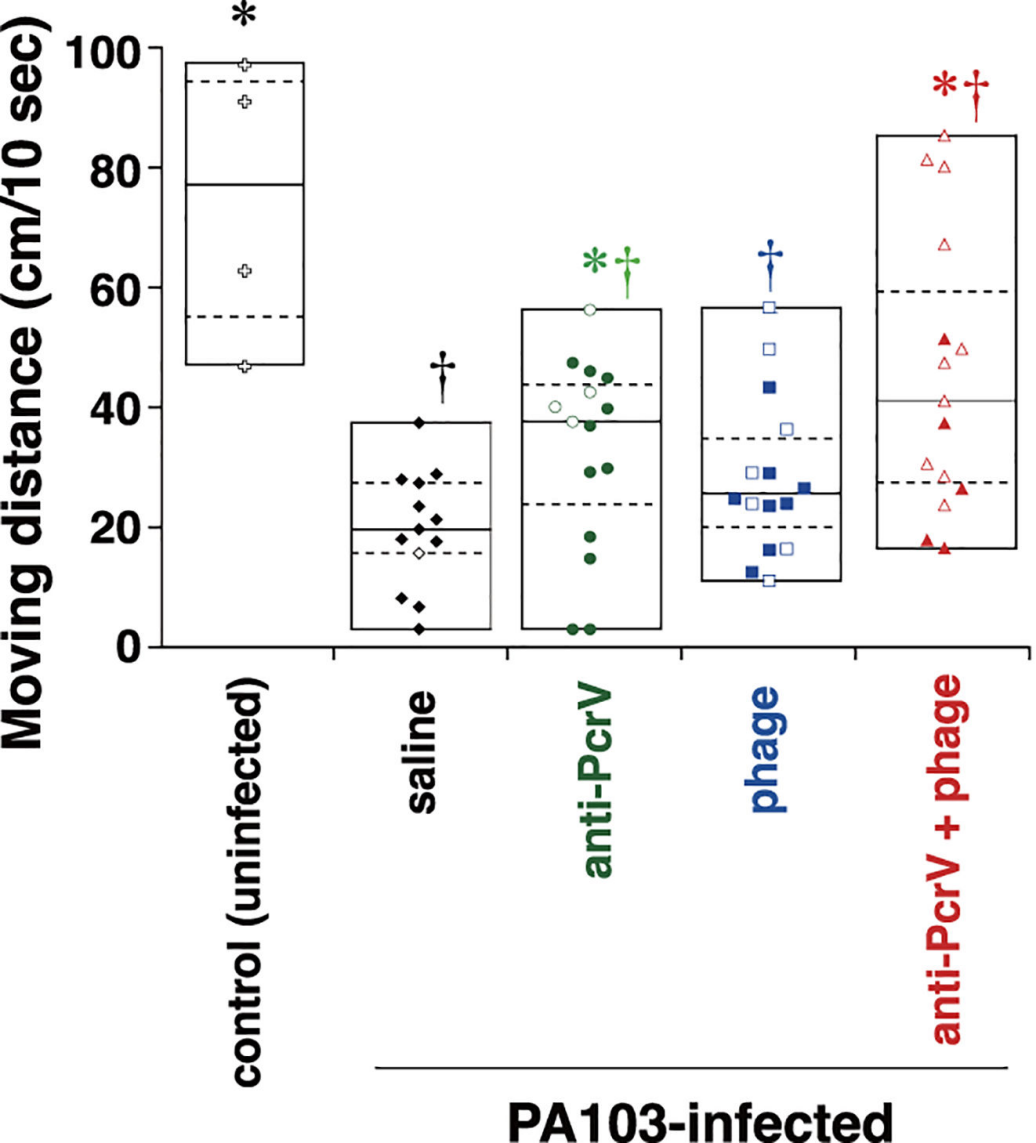
Activity test of infected mice. The movements of the mice in the cage for 10 s were evaluated 8 h after *P. aeruginosa* infection. The mice were divided into four groups that received the first instillation (either saline or a lethal dose of *P. aeruginosa* PA103) or the second instillation (either saline, anti-PcrV IgG, bacteriophages, or anti-PcrV IgG plus bacteriophages) as treatment. The data are presented as the median (a center solid bar in the box) and the first (dotted lines in the box) and second quartiles (bars), with each value indicated by a symbol (open symbol: survived, closed symbol: died). *P < 0.05 compared with the uninfected control group, †*P* < 0.05 compared with the saline group. The number of mice in each group is listed in Table 1.

The activity of the infected mice was quantitatively monitored 8 h after the infection (Fig. 4). While uninfected control mice moved around the cage at a median distance (25th–75th quartile) of 77.6 (61.0–92.0) cm/10 s, *P. aeruginosa* infection significantly decreased the activity to 19.7 (15.7–27.4) cm/10 s (†*P* = 0.015). Treatment with anti-PcrV and bacteriophages significantly maintained the activity at 37.7 (23.9–43.8) cm/10 s (*P* < 0.008). Mice in the phage group also tended to show better activity [24.8 (20.0–32.7) cm/10 s] than those in the saline group, but the difference was not significant (*P* = 0.058). However, the mice in the anti-PcrV and phage groups showed significantly improved activity [39.2 (27.5–59.3) cm/10 s] compared with that in the saline group (*P* = 0.008).

### Lung edema, bacteriological assays, myeloperoxidase activity, cytokine concentrations, and lung histology

**Fig 5:**
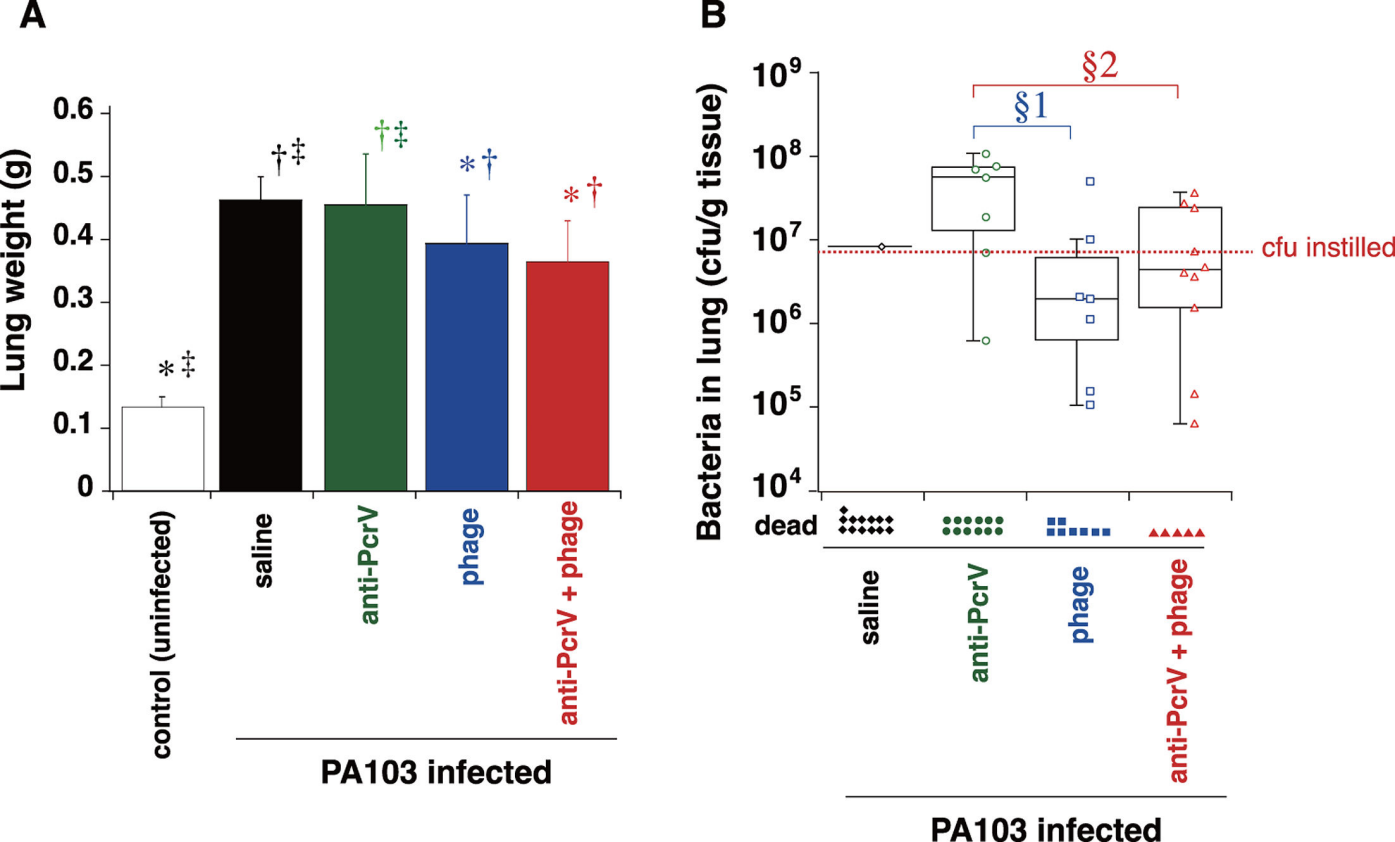
Lung edema and bacteriology of mice infected with *P. aeruginosa*. (A) Lung edema was evaluated as the wet lung weight (g) 24 h after intratracheal infection with *P. aeruginosa* PA103. The mice were divided into four groups that received the first instillation (saline or a lethal dose of *P. aeruginosa* PA103) or the second instillation (saline, anti-PcrV IgG, bacteriophages, or anti-PcrV IgG plus bacteriophages) as treatment. The data are presented as the mean ± SD. *P < 0.05 compared with the uninfected control group, †*P* < 0.05 compared with the saline group, ‡*P* < 0.05 compared with the anti-PcrV + phage group. (B) Bacterial CFU per gram of lung tissue in mice infected with *P. aeruginosa* and surviving for 24 h. Data are presented as the median (a center solid bar in the box) and the first (dotted lines in the box) and second quartiles (bars), with each value indicated by a symbol (open symbol: survived, closed symbol: died). §1*P* < 0.05, ratio below the initial bacterial dose (7.7 × 10^6^ CFU/g of normal lung tissue) compared with the phage group; §2*P* < 0.05, a ratio below the initial bacterial dose, compared with the anti-PcrV group. The number of mice in each group is listed in Table 1.

The severity of lung edema caused by the acute necrosis of lung epithelial cells due to the translocation of type III secretory ExoU toxin was evaluated as wet lung weight (gram) in mice (Fig. 5A). The mean wet lung weight of the uninfected control mice was 0.13 ± 0.02 g. In the mice infected with PA103, the lung weight was greater (0.46 ± 0.04 g) than that in the uninfected control mice (*P* < 0.01). Anti-PcrV treatment did not decrease the lung weight (0.46 ± 0.08 g). Anti-PcrV IgG with phages significantly reduced the lung weight to 0.39 ± 0.07 g and 0.36 ± 0.06 g, respectively (*P* = 0.038 and *P* = 0.001 vs saline-treated infected mice, respectively). Compared with anti-PcrV alone, anti-PcrV with phages significantly reduced the lung weight (*P* = 0.002).

Total lung bacterial counts were calculated for surviving mice 24 h after *P. aeruginosa* infection (Fig. 5B). Only one mouse in the saline group remained at 24 h. There was no significant difference in the CFU per gram of lung tissue among the three groups. However, in the anti-PcrV group, only one of the seven (14.3%) surviving mice had lower bacterial CFUs than the initial dose (1.0 × 10^6^ CFU/0.21 g of a normal lung). Five of the 7 (71.4%, *P* = 0.0001) surviving mice in the phage group and 7 of the 10 (70%, *P* = 0.0235) surviving mice in the anti-PcrV + phage group had lower bacterial CFUs than the initial dose. Therefore, treatment with both anti-PcrV and phages contributed to inhibiting the increase in bacteria after infection.

**Fig 6:**
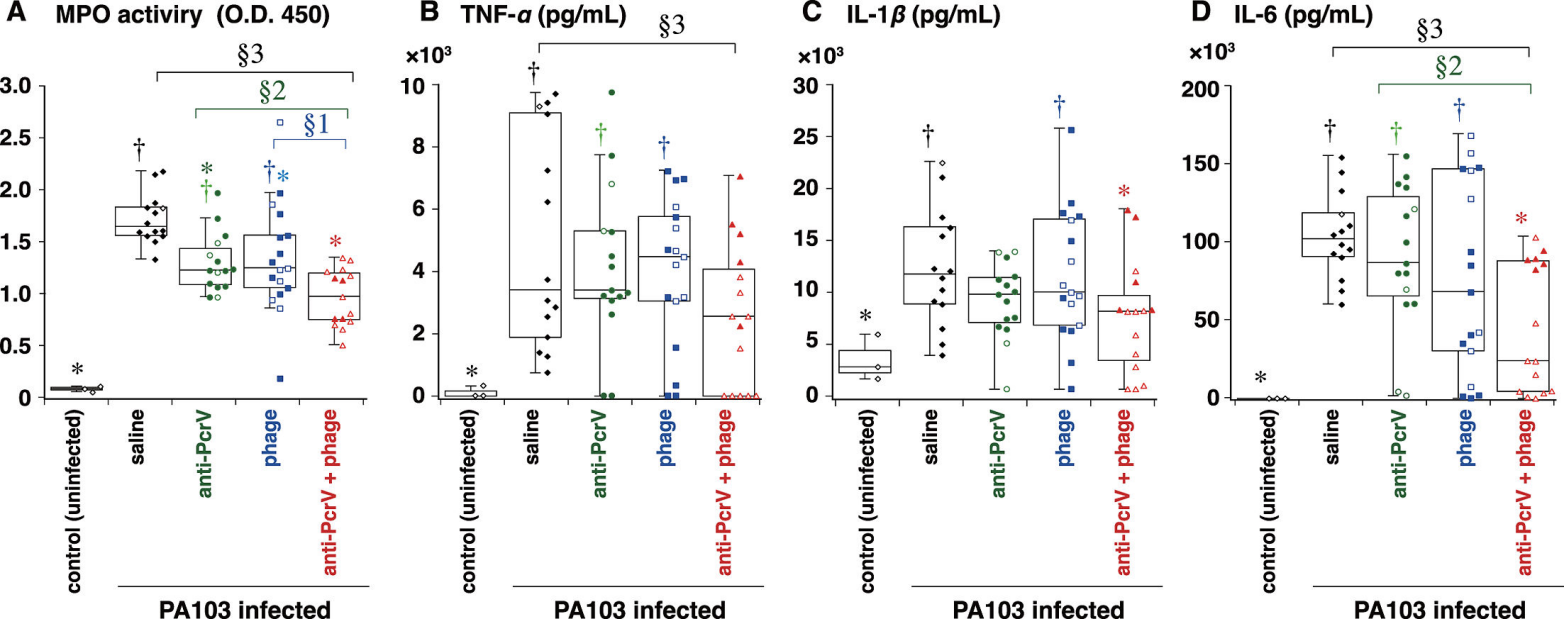
(A) MPO activity and cytokine concentrations in mouse lung homogenates. Mice first received a tracheal instillation of a lethal dose (1.0 10^6^ CFU/mouse) of *P. aeruginosa* PA103. (B–D) The lungs were then collected and homogenized 24 h after infection, and MPO activity and cytokine concentrations (TNF-α, IL-1β, and IL-6) in the lung homogenates were measured. The data are presented as the median (center solid bar in the box) and the first (boxes) and second (bars) quartiles, with each value indicated by a symbol (open symbol: survived, close symbol: died). †*P* < 0.05 compared with the uninfected control group, *P < 0.05 compared with the saline group; §1*P* < 0.05, comparison between the phage and anti-PcrV + phage groups; §2*P* < 0.05, comparison between the anti-PcrV and anti-PcrV + phage groups; §3*P* < 0.05, comparison between the saline and anti-PcrV + phage groups. IL, interleukin; TNF-α, tumor necrosis factor alpha.

To evaluate the severity of lung inflammation, neutrophil recruitment was quantitatively measured by myeloperoxidase (MPO) activity assay in lung homogenates at 24 h post-bacterial instillation (Fig. 6A). The mice that received bacteria intratracheally and were not treated had greater MPO activity in their lung homogenate than the uninfected control mice (*P* < 0.05). This MPO activity level was significantly lower in the anti-PcrV + phage group than in the phage or anti-PcrV groups (Fig. 6A) (*P* < 0.05).

The concentrations of inflammatory cytokines in the lung homogenates were measured (Fig. 6B through D). No increase in the cytokine concentration was detected in the uninfected control mice. Mice in the saline group had higher concentrations of tumor necrosis factor alpha (TNF-α), interleukin (IL)−1β, and IL-6 in their lungs than those in the uninfected control group (all *P* < 0.05). This increase in cytokine concentrations could not be suppressed by phage therapy alone or anti-PcrV therapy alone but was significantly attenuated by the combination of anti-PcrV and phage therapy (*P* < 0.05 vs the saline group) (Fig. 6B through D).

**Fig 7:**
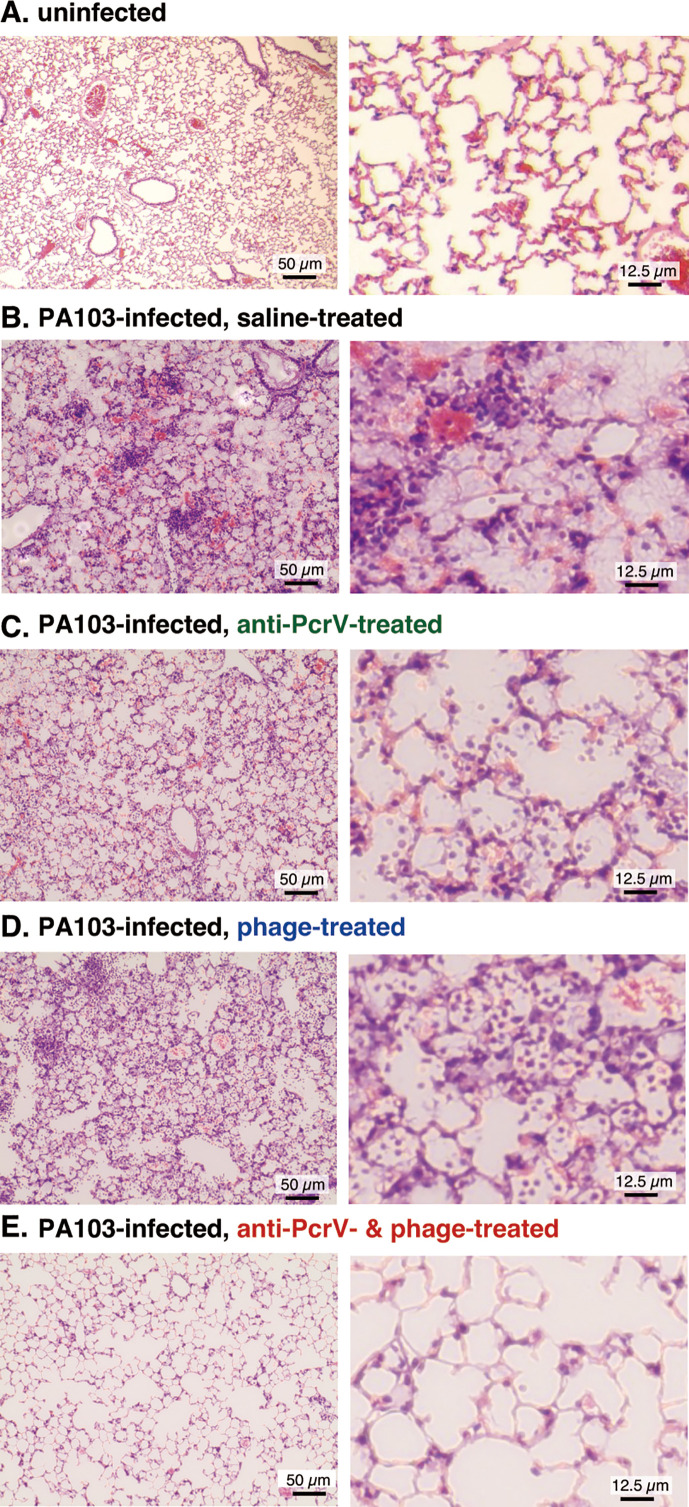
Lung histology of mice 24 h after infection with *P. aeruginosa*. The mice were divided into four groups that received the first instillation (either saline or a lethal dose of *P. aeruginosa* PA103) or the second instillation (either saline, anti-PcrV IgG, bacteriophages, or anti-PcrV IgG plus bacteriophages) as treatment. The lungs of the mice were fixed at 24 h post-infection. Hematoxylin–eosin staining was conducted after 10% formaldehyde fixation and paraffin embedding. (A) Uninfected control group, (B) saline group (infected mice), (C) anti-PcrV group (infected mice), (D) bacteriophage group (infected mice), and (E) anti-PcrV + phage group (infected mice). (Left side) ×200 magnification, scale bar = 50 µm; (right side) ×400 magnification, scale bar = 25 µm.

Histological changes were evaluated in the surviving mouse lungs 24 h post-infection. Enhanced neutrophil infiltration, alveolar hemorrhage, and destruction of the alveolar structure were observed in the lungs of mice in the saline group (Fig. 7). In contrast, the lungs of mice in the anti-PcrV group and phage group showed significantly fewer inflammatory changes than did those in the other groups. Almost no inflammatory changes were detected in the lungs of mice treated with either the anti-PcrV antibody or phages.

## DISCUSSION

*P. aeruginosa*, which has the largest genome among Gram-negative bacteria, is one of the major multidrug-resistant bacteria that already possess and can acquire various anti-microbial resistance mechanisms (1, 2). The emergence of multidrug-resistant *P. aeruginosa* strains, which cause various infections with high mortality rates due to resistance to different antibiotics, has enhanced the necessity of alternative and adjunct treatments other than antibiotics (1, 2). Since the 1990s, our research group and others have focused on the pathogenic mechanism by which *P. aeruginosa* causes acute lung injury at the molecular biological level (8, 9). These studies have shown that *P. aeruginosa* injects the bacterial type III secretory toxin phospholipase A_2_ toxin, ExoU, directly into target eukaryotic cells through the needle-like structure of the type III secretion system, causing acute pulmonary epithelial injury. In addition, we identified ExoU, a type III secretory toxin, as the most crucial lung-damaging factor involved in the type III secretion system (13). We also reported that the toxic effect of *P. aeruginosa* is due to the phospholipase A_2_ activity of ExoU (14–16). We found that a specific antibody against the PcrV protein, a cap-like structure located at the tip of the projection on the needle of the *P. aeruginosa* type III secretion apparatus, inhibits type III secretory toxicity and reduces acute lung injury (17). Therefore, in an *animal P. aeruginosa* lung injury model, acute lung epithelial injury occurs within 4 h after administering the *P. aeruginosa* cytotoxic strain PA103 expressing ExoU toxin (12, 13). Following the onset of pulmonary epithelial injury, bacteria diffuse deep into the body, causing bacteremia. Infected animals then rapidly fall into a state of septic shock because of the systemic spread of inflammatory mediators derived from infected lungs. ExoU translocation to pulmonary epithelial cells and alveolar macrophages leads to cell necrosis and acute lung injury (13). Therefore, in this model, some therapeutic interventions must be taken within 4 h to save the animals. This bacterial acute lung injury model, which involves administering a lethal dose of *P. aeruginosa* strain PA103 into the lungs that causes death within 24 h post-infection, is an extreme case compared to the pathophysiology of clinical infections. However, when isogenic mutant strains lacking the effective ExoU toxin (13, 14, 18)—a type III secretion toxin of *P. aeruginosa* or or PA103Δ*pcrV*, which lacks the *pcrV* gene crucial for the translocation of type III toxins (17)—are administered at the same bacterial load as the wild-type strain, neither acute lung injury nor mortality occurs in mice. In strains of *P. aeruginosa* lacking the *exoU* gene (such as strain PAO1), the lethal bacterial dose in the same animal model increased by more than 100 times (12). Additionally, infections caused by *P. aeruginosa* strains positive for the *exoU* genotype are frequently reported to be more severe clinically (19). Therefore, this *P. aeruginosa* acute lung injury model is considered adquate for evaluating whether some therapeutic intervention can suppress type III secretion toxicity.

In this animal model, pulmonary injury can be suppressed by increasing the titer of anti-PcrV antibodies, which can inhibit the type III secretion system, in infected host animals via active or passive immunization or antibody therapy (12, 20–22). A *P. aeruginosa* anti-PcrV monoclonal antibody has been developed for clinical use in humans and has been tested in phase II clinical trials (23–26). The inhibitory effect of the PcrV antibody on the type III secretion system is due to the binding of the antibody to PcrV and does not depend on the Fc portion of the antibody (20, 21). Anti-PcrV antibodies do not have bactericidal activity. Therefore, bacterial processing relies on the host animal’s phagocytic cells (e.g., alveolar macrophages and neutrophils) (17). Consequently, the inhibitory effect of the PcrV antibody on the type III secretion system can be rapid, but a healthy phagocytic cell system is a prerequisite.

Phage therapy is considered one of the possible approaches for treating *P. aeruginosa* infections (5, 27–31). However, despite the various benefits of bacteriophage therapy, there are also some disadvantages. In addition to the high specificity of individual phages to the bacterial species that phages infect, one disadvantage is that lysis takes a certain amount of time. Lysis time refers to the period spanning phage infection of a host cell and its lysis, at which point phage progenies are released (32). Under experimental conditions, lambda phage productivity is maximized around optimal lysis times ranging from 60 to 100 min, and the productivity of the lambda wild-type strain falls within this range (32–34). However, eukaryotic cell death induced by cytotoxic *P. aeruginosa* PA103 starts to occur within 1 h after infection (12). Therefore, phage therapy alone may not mitigate acute lung injury dependent on *P. aeruginosa* type III secretory toxicity, which starts occurring within 1 h of infection. In this study, we examined anti-PcrV therapy as a method other than the conventional anti-bacterial drug treatment method for treating *P. aeruginosa* infection, and it was expected to have an immediate inhibitory effect on type III secretory toxicity. We also examined the effect of bacteriophage therapy, which can subsequently demonstrate a bactericidal effect. Although our current findings detected a statistically significant difference in survival rates between anti-PcrV therapy alone and combined therapy, there were no statistically significant differences between the groups receiving phage therapy alone and combined therapy. However, the comprehensive results imply that combined therapy may be superior because of the significant disparities in survival rates between the untreated group, which received saline, and the combined therapy group, as well as notable differences in bacterial counts, MPO activity, and intrapulmonary IL-6 concentrations among the three therapeutic groups. These promising outcomes highlight the potential benefits of combined therapy and warrant further investigation. If this assumption is correct, acute lung injury caused by *P. aeruginosa* could be reduced more effectively, and the probability of subsequent bacteremia and septic shock could be reduced. We found that the combination of anti-PcrV therapy and bacteriophage therapy contributed to the suppression of pulmonary edema and bacterial growth and prevented mice from experiencing lethal septic shock.

In the amino acid sequence of *P. aeruginosa* PcrV, the central domain to which the anti-PcrV blocking antibody binds has been identified as a region with relatively few mutations (35, 36). However, bacteriophages are specific for each subspecies of bacteria. Therefore, screening is performed from a phage library to select appropriate phages that show effective anti-bacterial activity, or a phage cocktail containing suitable phages is used. Anti-type III secretory therapy with an anti-PcrV antibody and bacteriophage therapy have fast-acting bactericidal effects. This combination therapy has potential as an alternative therapeutic strategy to conventional anti-bacterial chemical agents.

One limitation of this study is that it evaluated an acute lung injury model using healthy mice even though *P. aeruginosa* typically infects immunocompromised hosts. In this healthy mouse model, even non-bactericidal anti-PcrV antibody therapy improved the condition to some extent, which may be attributed to the preserved function of neutrophils and macrophages in healthy mice. If the infected host is immunocompromised, leading to impaired phagocytic cell function, adjunct therapies such as bacteriophage therapy, which have bactericidal properties, might be more effective. In particular, it is of strong clinical interest whether repeated administration of antibodies or phages in an immunocompromised model can achieve sufficient therapeutic effects. Additionally, as another limitation of this study, the *P. aeruginosa* strain used in this study is a representative strain that secretes ExoU toxin. Past reports indicate that only approximately 20% of *P. aeruginosa* are ExoU positive, so similar effects may not necessarily be expected in infections caused by other strains, which make up nearly 80% of *P. aeruginosa* and are less toxic in terms of type III secretion (36). Bacteriophage therapy is still under development, and many aspects remain unknown, such as resistance acquisition by *P. aeruginosa* to phages and the long-term effects and side effects of repeated phage administration. It is undeniable that continuous research is essential for the clinical application of these findings.

The development of anti-PcrV monoclonal antibodies, derived from our mouse monoclonal antibody mAb166 (23–26), progressed to phase II clinical trials in approximately 2010 but was suspended due to cost-effectiveness and other development considerations. Further development of anti-PcrV antibodies from alternative pathways is also being pursued at the phase II level (37–40). Aside from neutralizing antibodies against toxin B, which causes *Clostridioides difficile*-associated pseudomembranous colitis, no monoclonal antibodies have reached clinical use for bacterial infections typically occurring in hospitals. Unfortunately, the development of monoclonal antibody therapies and targeted vaccines against pathogenic bacteria is stagnant due to factors such as development costs, the lethality of the conditions, and the risk of target antigen mutation (41). However, as drug resistance to conventional antibiotics continues to spread globally (42, 43), efforts to develop new treatments that do not rely on traditional antibiotics remain crucial. The combined therapy of anti-PcrV antibodies and phages is considered a potential pharmaceutical innovation, representing a future option for anti-infection treatments.

In conclusion, in a mouse model of *P. aeruginosa* pneumonia, combination therapy with an anti-PcrV antibody and bacteriophages reduced acute lung injury and improved survival, compared with each treatment alone. This combination therapy, which does not rely on conventional antibiotics, could constitute a new strategy for treating multidrug-resistant *P. aeruginosa* infections.

## MATERIALS AND METHODS

### Bacteriophage preparation

The *Pseudomonas* virus ΦR18 used in the present study was previously isolated from sewage water collected from a sewage treatment plant (Hokkaido, Japan) using plaque assays as previously described (7, 44). The whole genome of ΦR18 was also sequenced previously and submitted to the DDBJ/EMBL/GenBank databases under accession number LC102729.1. To perform downstream assays, ΦR18 was propagated by the plate lysate method as described by Nakamura et al. (45). An aliquot of the propagating strain Pa12 (46), grown in Luria–Bertani medium, was combined with an aliquot of ΦR18 and added to 3 mL of Luria–Bertani top agar containing 0.5% agarose ME (Iwai Chemicals Company, Tokyo, Japan). The mixture was overlaid on a Luria–Bertani agar plate. After overnight incubation of the plate at 37°C, 3 mL of SM buffer [10-mM MgSO_4_, 100-mM NaCl, 0.01% gelatin, and 50-mM Tris–HCl (pH 7.5)] was added to the plate, and the plate was incubated at room temperature for 1 h with shaking. The top agar overlay was scraped off and homogenized with SM buffer. The collected homogenate was centrifuged at 6,500 × *g* for 15 min at 4°C to remove the remaining bacteria and debris. The resulting supernatants were passed through 0.45-µm membrane filters (ADVANTC, Tokyo, Japan) and purified using Amicon Ultra-membrane filters (Merck, Darmstadt, Germany) according to the phage on tap method as described by Bonilla et al. (47). The phage titer was calculated as the number of plaques in a plaque assay employing the propagating strains, in accordance with a previous report (46), and is represented as PFU per milliliter.

### Monitoring the growth of PA103 with or without ΦR18

The lytic activity of ΦR18 was evaluated using turbidity assays with a plate reader, as previously reported (45). Briefly, phages were inoculated into cultures of PA103 in the mid-exponential phase (at MOIs of 1, 10, and 100), and the mixture was subsequently incubated at 37°C. The density of the culture was monitored at OD_590_ every hour for 24 h.

### *P. aeruginosa* strain, culture, and preparation

The *P. aeruginosa* PA103 strain, which was originally isolated from a patient in Australia in the late 1960s, was used in the challenge assay (48). This strain has a cytotoxic phenotype with positive type III secretion of ExoT and ExoU, and it carries the type III secretion system genotypes *exoS*−, *exoT*+, *exoU*+, and *exoY*+. However, this strain has an ExoY secretion-negative phenotype caused by a codon mutation. The culture of *P. aeruginosa* PA103 was grown at 32°C for 12 h in a shaking incubator and then centrifuged at 3,000 rpm for 10 min. The bacterial pellet was washed three times in saline and diluted to the appropriate number of CFU per milliter, as determined by spectrophotometry. The number of bacteria was confirmed by assessing the CFU of the diluted aliquot on a sheep blood agar plate.

### Mice

Certified pathogen-free male ICR mice (8–12 weeks old; body weight, 20–25 g) were obtained from Shimizu Laboratory Supplies Co., Ltd. (Kyoto, Japan). The mice were housed in groups of four to five individuals per cage with filter tops under pathogen-free conditions. The protocols for all animal experiments were approved by the Animal Research Committee of the Kyoto Prefectural University of Medicine (approval numbers: M2019-563, M2020-314, M2021-335, and M2022-326) prior to starting the experiments. Mouse housing was managed by the technical staff of the animal facility in our institution’s biosafety level 3 facility, and all animal experiments, including infection instillation and subsequent treatment and measurement, were conducted by us, the researchers, within the same facility.

### Anti-PcrV antibody

Endotoxin-free recombinant PcrV (rePcrV) was prepared as reported previously (49). In brief, the coding sequence for PcrV was amplified from the chromosome of *P. aeruginosa* PAO1 by polymerase chain reaction (PCR). The PCR fragments were ligated into an *Escherichia coli* expression vector (pQE30; Qiagen, Hilden, Germany) to create a protein construct with six tandem histidine residues (His-tags) at the amino terminus. The expression vector was introduced into the *E. coli* strain M15 (Qiagen), after which the recombinant proteins were produced from the *E. coli* culture and induced by isopropylthio-β-galactoside. The crude proteins were purified with nickel–nitrilotriacetic acid agarose columns (Novex R901-15; Thermo Fisher Scientific, Waltham, MA, USA), dialyzed for 72 h against phosphate-buffered saline (P3813; Sigma−Aldrich, St. Luis, MO, USA), and applied to columns made of endotoxin removal resin (Pierce, Thermo Fisher Scientific). The purity of rePcrV was evaluated using sodium dodecyl sulfate‒polyacrylamide gel electrophoresis, in which an intense single band appeared on the stained gel. The anti-PcrV polyclonal IgG fraction was then prepared from a rePcrV-vaccinated rabbit (Kitayama Labes, Ina, Japan), as previously reported (49).

### *P. aeruginosa* infection challenge in mice

A solution containing PA103 (1.0 × 10^6^ CFU in 60 µL of saline) was instilled into the lungs of each vaccinated mouse through an endotracheal needle, as described previously, under light inhalational anesthesia using sevoflurane (11). The survival and body temperatures of all the mice were monitored for 24 h, after which the survivors were euthanized. Notably, according to the infection experiment regulations of this facility, the experiment was completed within 2 h from the viewpoint of focusing on the pathogenesis of acute lung injury induced by *P. aeruginosa* in addition to animal welfare and preventing contamination within the facility.

### Lung edema, MPO activity, cytokine concentrations, and bacteriological assay

After the survivors were euthanized, the lungs of each mouse were collected, weighed, and homogenized using a homogenizer (Polytron PT10/35; Kinematica, Luzern, Switzerland) for further bacteriological evaluation. Lung edema due to acute lung injury induced by *P. aeruginosa* was evaluated in surviving and dead mice by measuring the wet weight of the lungs at the 24-h time point, as described previously (49). MPO activity in the lung homogenates was measured as reported previously (49). IL-1β, IL-6, and TNF-α concentrations in lung homogenates were quantified by an ELISA kit (BD Opt EIA Mouse IL-1β, IL-6, and TNF-α ELISA Sets, #559603, #555240, and #558534; BD Biosciences, Franklin Lakes, NJ, USA) according to the manufacturer’s instructions. The sequentially diluted lung homogenate was inoculated on a sheep blood agar plate and incubated at 37°C overnight to calculate the number of remaining bacteria in a gram of lung tissue.

### Activity test of infected mice

To evaluate the activity level of the mice, we recorded their movements in the cage for 10 s with a smartphone camera 8 h after *P. aeruginosa* infection. A sticker was attached to the back of the mouse, and the trajectory of the sticker was measured as the movement distance using the motion analysis software Kinobea (https://www.kinovea.org).

### Histopathological assay

A designated mouse in each group was euthanized for histological analysis at 24 h after infection. The lungs were perfused with 10% formalin neutral buffer solution for fixation and embedded in paraffin. The mounted sections were stained with hematoxylin–eosin.

### Statistical analysis

IBM SPSS statistical software (v.28.0.1.0; IBM Corp., Armonk, NY, USA) was used for the statistical analyses. KaleidaGraph (v.5.0.2; Synergy Software, Reading, PA, USA) was used for visualizing the data as graphs. Survival was assessed using Kaplan−Meier curves and log-rank tests. One-way analysis of variance and unpaired *t*-test were used to compare body temperature and lung weight, respectively, between the groups. The distance traveled and the number of bacteria in the lung were evaluated using the Kruskal−Wallis test and chi-square test, respectively. A *P* value of <0.05 was considered to indicate statistical significance. The statistical data for Fig. S3 to S6 are presented in the supplemental data in Table S1.

## SUPPLEMENTAL MATERIAL

Supplemental material spectrum.01781-24-s0001.xlsx Statistical data for each figure.

ASM does not own the copyrights to Supplemental Material that may be linked to, or accessed through, an article. The authors have granted ASM a non-exclusive, world-wide license to publish the Supplemental Material files. Please contact the corresponding author directly for reuse.
